# Metabolic Profiles Distinguish Non-Dampness-Phlegm and Dampness-Phlegm Patterns among Korean Patients with Acute Cerebral Infarction

**DOI:** 10.1155/2013/517018

**Published:** 2013-03-14

**Authors:** Min Ho Cha, A. Daniel Jones, Mi Mi Ko, Chen Zhang, Myeong Soo Lee

**Affiliations:** ^1^Medical Research Division, Korea Institute of Oriental Medicine, 1672 Yuseongdae-ro, Yuseong-gu, Daejeon 305-811, Republic of Korea; ^2^Department of Biochemistry and Molecular Biology, Michigan State University, 603 Wilson Road, East Lansing, MI 48824, USA; ^3^Department of Chemistry, Michigan State University, 578 South Shaw Lane, East Lansing, MI 48824, USA

## Abstract

Traditional Korean Medicine classifies stroke into four subtype patterns according to symptomatic pattern identification: Qi deficiency (QD), Yin deficiency (YD), Dampness-phlegm (DP), and Fire and Heat (FH). This study investigated the difference in metabolic profiles of plasma comparing subjects displaying non-DP and DP patterns. A total of 141 patients with cerebral infarction enrolled in this study were distributed as non-DP (*N* = 68) and DP (*N* = 73). Anthropometric parameters and symptom/sign index were measured. Metabolic profiling was performed using ultrahigh-performance liquid chromatography-mass spectrometry. The Ratio of subjects with slippery pulse was higher in DP pattern, but fine pulse was lower than that in non-DP pattern. As a result of metabolomics analysis, twenty-one metabolites displayed different levels between non-DP and DP patterns. Two were identified as lysophosphatidylcholines (LPCs), LPC(18:2), and LPC(20:3) having an unsaturated acyl chain and showed lower levels in DP pattern than in non-DP pattern (*P* = 0.015, 0.034, resp.). However, the saturated LPCs, LPC(18:0) and LPC(16:0), exhibited slight but statistically insignificant elevation in DP pattern. Our results demonstrated that plasma LPCs with polyunsaturated fatty acid groups were associated with DP pattern and suggest that variation of plasma lipid profiles may serve as potential biomarker for diagnosis of DP pattern.

## 1. Introduction

Pattern Identification (PI) is a unique diagnosis system of traditional medicine practiced in East Asian countries including China, Korea, and Japan and provides information that guides appropriate treatment of patient disease. Previous reports described how traditional Korean medicine (TKM) categorizes stroke as four patterns according to their symptoms and sign: Qi deficiency (QD), Dampness-phlegm (DP), Yin deficiency (YD), and Fire and Heat (FH) [1]. Among them, the DP pattern is attributed to the prevention of Qi-blood circulation in the body, resulting in a pathological product [2]. Most notably, the DP pattern is associated with overweight/obesity and dyslipidemia [3–6]. Kim et al. reported that body mass index (BMI) of subjects with DP pattern was significantly increased compared with subjects exhibiting other patterns [5], and Kang et al. showed that the levels of total cholesterol, LDL-cholesterol and triglycerides in serum of subjects displaying the DP pattern were also higher than subjects with non-DP pattern [6].

However, the diagnosis of PI depends on symptoms/sign exhibited by patients, which may be interpreted incorrectly. Therefore, it is necessary to establish scientific evidence to aid accurate diagnosis based on PI. Previously, several studies showing relation between biological factors and PI were reported [7–13]. In particular, NPY and UCP2 and PON1, related with obesity and dyslipidemia, were significantly associated with DP pattern in Korean stroke patients [8, 9, 13]. 

Metabolomics provides a powerful approach for global and quantitative assessment of endogenous small molecule metabolites within biological fluids [14] and has been utilized to propose candidate biomarkers of several diseases [15–20]. Metabolites are the small chemical compounds or products involved in various metabolic pathways, and the changes of their levels serve as indicators of changes of pathology or physiology [14]. Recently, metabolomics investigations were performed to explain PI of rheumatoid arthritis and type 2 diabetes in Chinese populations [21, 22]. 

In this study, we analyzed metabolic profiles in plasma of acute patients with cerebral infarction (CI) by ultrahigh-performance liquid chromatography/time-of-flight mass spectrometry (UHPLC-TOFMS) and searched for metabolites differing in levels between DP and non-DP patterns. 

## 2. Materials and Methods

### 2.1. Study Subjects

This study was collected as parts of the project “The Fundamental Study for the Standardization and Objectification of PI in TKM for Stroke (SOPI-Stroke)” in the Korean Institute of Oriental Medicine (KIOM) [23]. 

Patients with CI were admitted from 2009 to 2010 to the following Korean oriental medical hospitals participating in this study: Kyung Hee Oriental Medical Center (Seoul), Kyung Hee East-West Neo Medical Center (Seoul), Dong Guk International Hospital (Kyunggi-do), and Dae Jeon Oriental Medical Hospital (Daejeon). Subjects had to be enrolled as CI patients within three days of the onset, and their symptoms were confirmed by imaging diagnosis, using computerized tomography (CT), magnetic resonance imaging (MRI), or magnetic resonance angiography (MRA). CI subtypes were classified as large artery atherosclerosis (LAA), cardioembolism (CE), small vessel occlusion (SVO), stroke of other determined etiology (SOE), and stroke of undetermined etiology (SUE) subtype according to the trial of ORG 10172 in the Acute Stroke Treatment (TOAST) classification. Subjects with subarachnoid, subdural, or epidural hemorrhage were excluded in this study, and subjects with diabetes mellitus and stroke in history and with infectious diseases and liver diseases were also excluded. 

After obtaining written informed consent from all subjects, clinical data were collected. This study was approved by the Institutional Review Boards (IRB) of the KIOM and by each of the oriental medical hospitals. 

### 2.2. PI Diagnosis of CI Patients

The symptoms and signs of subjects were collected using “stroke PI case report form” [2], and PI diagnosis of subjects was determined by two expert TKM doctors based on “Korean Standard PIs for Stroke-III” previously reported by Lee et al. [1]. Subjects receiving differing diagnoses from the two doctors were excluded. The number of patients was 68 in non-DP and 73 in DP.

### 2.3. Plasma Preparation

Preparation of plasma from each subject for UHPLC-MS analysis was performed according to protocols provided by Human Serum Metabolome (HUSERMET) Consortium [24]. Briefly, 200 *μ*L of plasma thawed on ice was thoroughly mixed with 600 *μ*L methanol and 100 *μ*L of internal standard solution and centrifuged at 15000 g for 15 min at 25°C. Supernatant was transferred and completely dried by using centrifugal vacuum evaporator over 4 hours. Dried samples were resolved by adding 100 *μ*L of melting solution (acetonitrile/isopropanol/water, 3 : 2 : 2) and centrifuged at 15000 g for 15 min at 25°C. Supernatant was transferred into vial and used for UHPLC-MS analysis. 

### 2.4. Metabolic Profiling Analysis by UHPLC-MS

A 10 *μ*L aliquot of the concentrated plasma extract was injected into an Ascentis Express C18 column (10 cm × 2.1 mm, 2.7 *μ*m particles: Supelco, Bellefonte, PA, USA) using a SIL-5000 autosampler (Shimadzu, Tokyo, Japan). The solvent was delivered by LC-20AD pumps (Shimadzu Co., Tokyo, Japan) at a flow rate of 0.4 mL/min, with solvent A being 0.15% aqueous formic acid and solvent B being acetonitrile/isopropanol (2 : 1). Gradient elution was performed and equilibrated with water containing 0.15% formic acid. Samples were eluted by gradient containing 0.15% formic acid for 30 min, and metabolites separated by UHPLC were assigned by MS (Waters, Milford, MA, USA). Separate injections were made for analyses conducted ESI-positive and ESI-negative modes. 

The voltage of capillary and sampling cone was, respectively, set at 3 kV and 10 V, and the desolvation flow was set to 350 L/h at a temperature of 350°C. The source temperature was set to 100°C. Collecting range of MS data was *m*/*z* 100–2000 with a scan time of 0.2 s. The accurate mass and composition for the metabolite ions were calculated by MassLynx 4.1 (Waters, Milford, MA, USA) incorporated in the instrument.

### 2.5. Data Processing and Identification of Metabolites

Results from UHPLC-MS analyses were processed using automated peak detection, integration, and retention time alignment using MarkerLynx 4.1 software (Waters, Milford, MA, USA), and MS data were assembled into a matrix of integrated extracted ion chromatogram peak areas, organized by mass and retention time. Parameters for MarkerLynx analysis were set as follows: peak width at 5% height: 15 s, intensity threshold: 20, mass window: 0.1 amu, retention time window: 0.5 min, noise elimination level: 6, mass tolerance: 0.1 Da. Normalization of MS spectra was performed by total normalization, and peaks not detected from over 2% of subjects were excluded in the following analysis. Metabolites were annotated using searches of the ChemSpider database (http://www.chemspider.com/) and Human Metabolome Database Version 2.5 (http://www.hmdb.ca/) or/and confirmed by external standards based on retention time and mass spectra.

### 2.6. Statistical Analysis

The statistical analysis of our data was performed with IBM SPSS Statistics 19 (IBM Co., New York, NC, USA). Normality of continuous variables in clinical data was decided by Kolmogorov-Smirnov test, and statistical difference was determined by *t*-test in parametric variable or Mann-Whitney *U* test in nonparametric variable. Categorical variables were compared with a chi-squared test or Fisher's exact test. Results of MS analysis were represented as the mean ± SD, and the difference in mass intensity of plasma metabolites between the two groups was tested by independent *t*-test with Mann-Whitney *U* test. The statistical significance was set at *P* < 0.05. 

## 3. Results

The general characteristics between non-DP and DP were shown in Table 1. The mean age of the DP pattern patients was slightly higher than that of the non-DP pattern patients. BMI in body composition and total cholesterol and LDL-cholesterol in serum parameters also showed higher levels in the DP pattern patients, which was similar to previous reports by Kim et al. and Lim et al. [3, 13]. The distribution of symptoms and signs according to “Korean Standard PIs for Stroke-III,” which are parameters to decide the pattern of stroke [1], is shown in Table 2. Among pulse diagnoses, the ratio of subjects with slippery pulse in DP pattern was significantly higher (54.8%) than that in non-DP pattern (38.2%) (*P* = 0.0489). In contrast, the fraction of subjects exhibiting weak pulse was significantly lower in DP pattern (*P* = 0.0073). 

**Figure 1:**
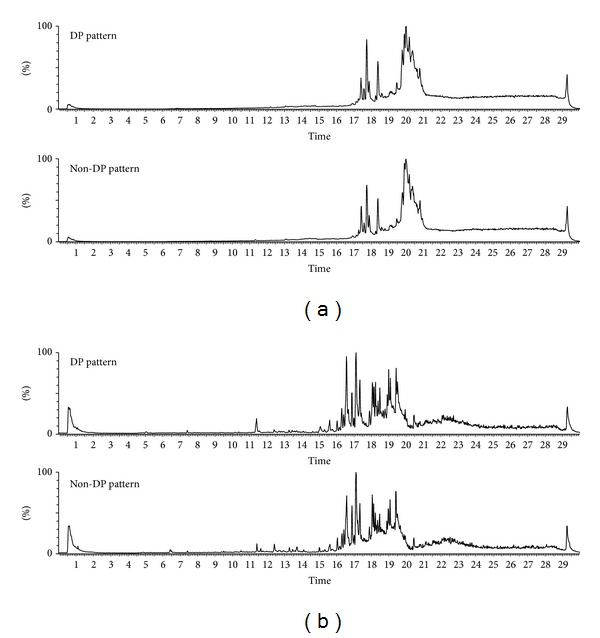
Chromatographic pattern of human plasma metabolite in ESI-positive (a) and ESI-negative mode (b) generated using UHPLC-MS analysis.

To find metabolite levels in plasma that could differentiate non-DP and DP patterns, we performed UHPLC-MS analysis (Figure 1). A list of metabolites present in significantly different levels in plasma of non-DP and DP patterns is presented in Table 3. Three abundant metabolites, detected as ions of *m*/*z* 520.366, 1039.724, and 1043.763, in positive-ion mode and *m*/*z* 590.335 in negative-ion mode were significantly lower in the DP pattern relative to the non-DP pattern. However, most differentiating metabolites were present at much lower levels or were removed by the abundance threshold of 20 ion counts. 

To identify each differentiating metabolite, public databases were searched using ion masses determined using both positive-ion and negative-ion modes. Phosphatidylcholine (PC) and lysophosphatidylcholine (LPC) lipids were identified from positive-ion mode data (Table 4). For example, the mean normalized peak areas of LPC (18 : 2; 9Z, 12Z) and LPC (20 : 3), detected as *m*/*z* 520.366 and 546.399, were 282 and 35 in DP pattern and were significantly lower than 304 and 41 in non-DP pattern (*P* = 0.010 and *P* = 0.034, resp.). Additional LPCs with unsaturated fatty acid, LPC (20 : 4) and LPC (18 : 1), also showed a tendency to be lower in DP than non-DP pattern, but without significance (*P* = 0.070 and *P* = 0.77, resp.). Otherwise, levels of LPCs without double bond, LPC (14 : 0), LPC (16 : 0), and LPC (18 : 0), were not different between two groups. 

In the negative-ion mode data, a metabolite detected as *m*/*z*  590.335 was significantly lower in DP pattern (*P* = 0.017) and was identified as the formate ion adduct of LPC (20 : 3), confirming the result from positive-ion mode. Free fatty acids (FFA) and lysophosphatidylethanolamine (LPE), which were mainly detected in negative-ion mode analyses, were not different in the two patterns (Table 5). Only LPE(18:2) showed a tendency to be lower in the DP pattern (*P* = 0.065).

## 4. Discussion

PI presents a comprehensive system for the diagnosis of disease and is based on the patient's signs and symptoms. TKM doctors determine the cause, nature, and treatment of the illness depending on signs and symptoms in patients and use this information to provide individualized treatment to patients, who have the same disease [3]. This approach is similar to what has been described as personalized medicine, which has recently emphasized that patients should receive different treatments according to the patient's individual condition to increase the positive effect of treatment and reduce adverse effects [25, 26]. But general concepts between PI and personalized medicine are different. PI focuses on the signs and symptoms in patients to define a phenotype that guides treatment selection. Much of the emphasis of modern personalized medicine is based on the genetic background, or genotype, of each patient [3, 27]. Therefore, studies to establish relationships between PI and personalized medicine have been performed [10, 11, 13]. 

In TKM, stroke is classified into four standard subtypes according to PI: QD, DP, YD, and FH based on position, internal or external etiology, or observations of affected organ. DP, which is classified by etiology, is a combination of phlegm and internal dampness causing disease. Major symptoms of the DP pattern include anorexia, dyspepsia, dizziness, headache, and heaviness [28]. However, not all patients with DP pattern have all of these symptoms, and subjects with other patterns often exhibit some of the same symptoms. For example, only 15% of subjects had dizziness with nausea, considered a major indicator in the DP pattern, and this ratio was not different in subjects with non-DP pattern. Slippery pulse, which is an important factor for the diagnosis of DP pattern and significantly high ratio at DP pattern, occurred in the non-DP pattern (Table 2). These complexities of sign and symptoms complicate PI diagnosis and may cause diagnostic error between TKM doctors. Previous studies reported that concordance rates between two expert TKM doctors about PI diagnosis for stroke, pulse, or tongue diagnosis were 70–90%, which means that the diagnostic error may always be implied [29–31]. For these reasons, reliable biomarkers for more accurate diagnosis of PI are needed. 

Metabolomics provides powerful tools to measure and identify metabolic biomarkers from biofluids, and earlier studies to explain PI using the change in metabolite levels were performed in China [21, 22]. In this study, we elucidated UHPLC-MS analysis from plasma of the subjects with CI to find metabolites related with DP pattern of stroke (Figure 1). From the total of 612 peaks corresponding to specific mass-retention time pairs, 21 metabolite peaks showed significant differences in the levels between DP and non-DP patterns (Table 3). In addition, searches of open metabolome databases allowed annotation of 57 major metabolites detected using this protocol (Tables 4 and 5). The primary conclusions are that the levels of LPC (18 : 2, 9Z, 12Z) and LPC (20 : 3) were significantly lower in DP pattern than non-DP pattern (*P* = 0.010 and 0.034, resp.), and LPC (20 : 4) and LPC (18 : 1) showed a tendency to decrease in DP pattern (*P* = 0.070 and *P* = 0.077). 

Lysophosphatidylcholines (LPCs), called lysolecithins, are a class of phospholipids derived from phosphatidylcholines and produced by two pathways. First is the result from partial hydrolysis of phosphatidylcholine, so that one of the fatty acids is removed by the action of phospholipase A2 [32]. A second pathway for LPC formation occurs by the transfer of one fatty acid of phosphatidylcholine to cholesterol by lecithin-cholesterol acyltransferase (LCAT), which is an enzyme that converts free cholesterol into cholesteryl ester. Cholesteryl ester is a more hydrophobic form of cholesterol that is sequestered into the core of a lipoprotein particle [33] and into liver. 

Until now, relationships between plasma levels of LPCs and DP pattern were not known, but earlier reports suggested the possibility of such correlations. It was known that DP pattern was related to obesity and hyperlipidemia [3, 8, 9], and some metabolomic analyses showed that plasma LPC levels were also associated with obesity [34–37]. Barber et al. and Kim et al. reported that plasma levels of LPCs with one or two double bonds such as lyso (16 : 1), LPC (18 : 1), LPC (18 : 2), and LPC (20 : 1) were significantly reduced in high-fat diet-induced obese mice [35, 36], In another study among overweight/obese subjects performed by Kim et al., LPC (18 : 1), LPC (18 : 2), and LPC (20 : 2) were decreased in the plasma of overweight/obese subjects [37]. Of particular note in these studies was the saturated LPC (18 : 0), that increased in obese subjects and obese mice model [36, 37], and these results parallel the findings in the current investigation (Table 4). 

This study showed, for the first time, that some LPCs in plasma were associated with DP pattern in CI patient population. However, this study included several limitations. First, this is a simple cross-sectional study, and the subjects enrolled in this study were very acute patients. Second, this study does not have a sufficient sample size to generalize the relationship between plasma metabolites and DP pattern. Third, the PI was limited to stroke diagnoses. The lack of evidence on the accuracy of PI forced us to perform an observational study, not allowing a randomized controlled trial. Despite of these limitations, this study provided metabolic information about DP patterns, and further studies should be performed in subjects of another large population to generalize the conclusions of this study. 

## Figures and Tables

**Table 1:** General characteristics of subjects.

Characteristics	Non-DP	DP	*P*
*N*	68	73	
Gender (M/F)	38 (55.9)/30 (44.1)*	36 (49.3)/37 (50.7)	0.501
Age (year)	67.50 ± 11.43	69.73 ± 11.07	0.213^b^
TOAST (LAA/CE/SVO/SOE/SUE)	15 (22.1)/1 (1.5)/43 (63.2) /3 (4.4)/6 (8.8)	17 (23.3)/4 (5.5)45 (61.6) /0 (0.0)/7 (9.6)	0.300
Weight (kg)	63.18 ± 11.49^#^	63.67 ± 10.63	0.799^a^
BMI (kg/m^2^)	24.40 ± 3.75	24.43 ± 3.50	0.968^a^
Smoking (none/strop/active)	33 (48.5)/11 (16.2)/24 (35.3)	36 (49.3)/7 (9.6)/30 (41.1)	0.470
Drinking (none/stop/active)	21 (30.9)/11 (16.2)/36 (52.9)	25 (34.2)/15 (20.5)/33 (45.2)	0.632
History disease			
TIA	7 (10.3)	8 (11.1)	1.000
Hypertension	37 (54.4)	36 (49.3)	0.614
Hyperlipidemia	6 (8.8)	7 (9.6)	1.000
Heart disease	4 (5.9)	2 (2.8)	0.429
Serum parameters			
GOT (U/dL)	23.51 ± 9.25	22.97 ± 6.13	0.666^b^
GPT (U/dL)	20.26 ± 10.10	20.52 ± 8.50	0.623^b^
Total cholesterol (mg/dL)	193.66 ± 41.89	197.14 ± 43.83	0.632^a^
Triglyceride (mg/dL)	192.51 ± 119.82	187.63 ± 172.77	0.176^b^
HDL-cholesterol (mg/dL)	42.97 ± 10.64	43.34 ± 10.47	0.735^b^
LDL-cholesterol (mg/dL)	113.18 ± 35.32	119.25 ± 38.19	0.344^a^
Blood sugar (mg/dL)	116.46 ± 33.68	117.21 ± 39.63	0.835^b^

*indicates the number of subjects (%), and ^#^indicates the value of mean ± SD. The *P* value of categorized variables was calculated by chi-squared test or Fisher's test, and continuous variables were analyzed by a student's *t*-test (a) or Mann-Whitney *U* test (b) after a normality test and compared with the non-DP group. DP: Dampness-phlegm, BMI: body mass index, TIA: transient ischemic attack, GOT: glutamic oxaloacetic transaminase, GPT: glutamic pyruvate transaminase.

**Table 2:** Difference distribution of symptoms/signs between Dampness-phlegm and non-Dampness-phlegm.

Symptoms and signs	Non-DP (*n* = 68)	DP (*n* = 73)	*P*
Vexation and insomnia	14 (20.59)	9 (12.33)	0.1847
Reddened complexion	27 (39.71)	28 (38.36)	0.8696
Blood-shot eyes	8 (11.76)	10 (13.7)	0.731
Wheezing in the throat with sputum	14 (20.59)	18 (24.66)	0.5643
Fetid mouth odor	15 (22.06)	12 (16.44)	0.3967
Thirst	26 (38.24)	24 (32.88)	0.5063
Red tongue	28 (41.18)	25 (34.25)	0.3959
Yellow fur	13 (19.12)	21 (28.77)	0.1808
Thick fur	23 (33.82)	36 (49.32)	0.0624
Heat vexation in the chest	14 (20.59)	12 (16.44)	0.5255
Turbid urine	8 (11.76)	14 (19.18)	0.2255
Rapid pulse	17 (25)	19 (26.03)	0.8888
Strong pulse	26 (38.24)	33 (45.21)	0.4018
Surging pulse	7 (10.29)	4 (5.48)	0.2868
Heat vexation and aversion to heat	20 (29.41)	21 (28.77)	0.9329
Heat in the palms and soles	7 (10.29)	5 (6.85)	0.4639
Vexing heat in the extremities	4 (5.88)	3 (4.11)	0.7113
Pale face and red zygomatic site	13 (19.12)	6 (8.22)	0.0583
Dry mouth	40 (58.82)	33 (45.21)	0.1059
Dry fur	10 (14.71)	19 (26.03)	0.0965
Bare and red tongue like a mirror	4 (5.88)	2 (2.74)	0.4289
Night sweating	5 (7.35)	9 (12.33)	0.3235
Tidal fever	4 (5.88)	2 (2.74)	0.4289
Thin	14 (20.59)	17 (23.29)	0.6989
Drowsiness, likes to lie down	14 (20.59)	10 (13.7)	0.2767
Feels powerless and lazy	30 (44.12)	28 (38.36)	0.4872
Looks powerless and lazy	27 (39.71)	29 (39.73)	0.9981
Pale complexion	6 (8.82)	5 (6.85)	0.6623
Reluctance to speak	6 (8.82)	14 (19.18)	0.0782
Pale tongue	14 (20.59)	12 (16.44)	0.5255
Teeth-marked tongue	8 (11.76)	9 (12.33)	0.9181
Slow pulse	7 (10.29)	13 (17.81)	0.2013
Weak pulse	10 (14.71)	10 (13.7)	0.8640
Fine pulse	9 (13.24)	1 (1.37)	0.0073
Reversed cold in the extremities	7 (10.29)	7 (9.59)	0.8887
Sallow complexion	20 (29.41)	23 (31.51)	0.7872
Dark inferior palpebral	14 (20.59)	17 (23.29)	0.6989
Dizziness with nausea	11 (16.18)	11 (15.07)	0.8562
White fur	42 (61.76)	49 (67.12)	0.5063
Enlarged tongue	12 (17.65)	10 (13.7)	0.5185
Slippery pulse	26 (38.24)	40 (54.79)	0.0489
Heavy	27 (39.71)	26 (35.62)	0.6164

Data presented the number of subjects (%) with symptoms/signs. *P* value was calculated by chi-squared test or Fisher's test.

**Table 3:** List of metabolites exhibiting different levels in plasma between Dampness phlegm and non-Dampness phlegm.

Retention time (min)	Mass (*m/z*)	Non-DP	DP	*P*
ES (+) mode				
20.04	1461.2680	6.61 ± 1.43	7.38 ± 2.09	0.009
17.18	1039.724	153.13 ± 44.87	134.44 ± 52.43	0.010
17.15	520.3663	304.62 ± 48.29	282.22 ± 53.29	0.015
20.27	1494.291	18.50 ± 4.16	20.03 ± 4.99	0.022
17.3	502.3353	15.82 ± 5.11	14.21 ± 4.05	0.024
17.46	546.3991	41.14 ± 17.33	35.35 ± 14.02	0.034
19.71	1376.208	20.36 ± 6.07	23.05 ± 8.81	0.037
18.35	269.2497	4.13 ± 1.26	3.66 ± 1.15	0.042
17.69	1043.763	113.36 ± 46.83	96.79 ± 36.09	0.044
21.13	237.1001	26.71 ± 11.22	30.31 ± 10.32	0.046
17.68	1565.1790	4.15 ± 2.49	3.33 ± 1.53	0.047
29.01	195.0539	5.93 ± 1.88	6.57 ± 1.97	0.048
ES (−) mode				
17.14	1019.5881	10.58 ± 5.27	8.77 ± 5.09	0.010
0.6	444.6674	1.32 ± 0.95	1.55 ± 0.82	0.013
0.53	740.8311	5.84 ± 2.06	6.63 ± 2.25	0.015
17.5	590.3351	104.35 ± 42.97	88.28 ± 38.06	0.017
29.23	599.3050	2.50 ± 0.82	2.20 ± 0.69	0.015
29.23	288.1364	3.15 ± 1.06	3.47 ± 1.01	0.029
0.53	462.8737	1.23 ± 0.49	1.44 ± 0.59	0.032
17.53	658.3196	4.30 ± 1.33	3.89 ± 0.98	0.038
19.87	961.5879	7.01 ± 3.20	8.21 ± 3.71	0.042

Data are presented as mean normalized peak areas ± SD. The *P* value was calculated by Mann-Whitney *U* test.

**Table 4:** Identification and quantification of metabolites in human plasma using UHPLC-MS and electrospray ionization in positive-ion mode.

Retention time (min)	Common name	Formula of [M+H]^+^	Measured mass (*m/z*)	Theoretical mass (*m/z*)	Mass error (Da)	Non-DP	DP	*P*
16.55	LPC(14:0)	C_22_H_47_NO_7_P^+^	468.35	468.31	+0.04	21.87 ± 11.51^a^	22.95 ± 9.12	0.145
16.86	LPC(16:1 (9Z))	C_24_H_49_NO_7_P^+^	494.36	494.32	+0.04	58.80 ± 35.99	56.53 ± 27.33	0.671
17.08	LPC(22:6)	C_30_H_51_NO_7_P^+^	568.38	568.34	+0.04	75.58 ± 30.32	77.35 ± 37.30	0.961
17.12	LPC(20:4)	C_28_H_51_NO_7_P^+^	544.37	544.34	+0.03	104.65 ± 31.01	94.62 ± 33.85	0.070
17.15	LPC(18:2(9Z, 12Z))	C_26_H_51_NO_7_P^+^	520.36	520.34	+0.02	304.62 ± 48.29	282.22 ± 53.29	0.010
17.15	unknown		1063.75			45.49 ± 17.50	40.35 ± 17.42	0.058
17.46	LPC(20:3)	C_28_H_53_NO_7_P^+^	546.39	546.35	+0.04	41.14 ± 17.33	35.35 ± 14.02	0.034
17.51	LPC(16:0)	C_24_H_51_NO_7_P^+^	496.36	496.34	+0.02	530.37 ± 56.28	533.84 ± 64.59	0.583
17.69	LPC(18:1)	C_26_H_53_NO_7_P^+^	522.38	522.36	+0.02	261.34 ± 38.55	249.65 ± 39.38	0.077
18.21	LPC(18:0)	C_26_H_55_NO_7_P^+^	524.39	524.37	+0.02	398.11 ± 41.16	402.10 ± 52.47	0.391
19.10	Dioctyl phthalate (plasticizer contaminant)	C_24_H_39_O_4_^+^	391.31	391.28	+0.03	31.27 ± 5.95	31.27 ± 4.95	0.817
19.38	DG(40:4)	C_43_H_77_O_5_^+^	673.58	673.58	+0.007	32.77 ± 15.44	36.78 ± 19.95	0.306
19.67	DG(40:3)	C_43_H_79_O_5_^+^	675.58	675.59	-0.01	228.01 ± 38.68	229.73 ± 45.96	0.812
19.73	DG(42:4)	C_45_H_81_O_5_^+^	701.59	701.60	−0.01	289.45 ± 45.60	289.82 ± 44.59	0.961
19.85	PC(36:5)	C_44_H_79_NO_8_P^+^	780.59	780.55	+0.04	213.70 ± 45.71	224.66 ± 45.41	0.156
19.90	PC(34:3)	C_42_H_79_NO_8_P^+^	756.61	756.55	+0.06	57.74 ± 29.96	58.82 ± 28.32	0.840
19.95	PC(38:6)	C_46_H_81_NO_8_P^+^	806.61	806.56	+0.05	219.88 ± 37.58	228.46 ± 45.82	0.501
19.99	DG(42:3)	C_45_H_83_O_5_^+^	703.61	703.62	−0.01	172.78 ± 29.19	179.24 ± 41.33	0.564
20.00	Unknown		1485.24			63.66 ± 25.34	66.75 ± 23.35	0.536
20.01	PC(36:4)	C_44_H_81_NO_8_P^+^	782.62	782.57	+0.05	131.29 ± 18.25	128.28 ± 22.48	0.095
20.08	PC(38:5)	C_46_H_83_NO_8_P^+^	808.66	808.59	+0.07	30.93 ± 11.17	32.02 ± 13.12	0.760
20.09	PC(34:2)	C_42_H_81_NO_8_P^+^	758.60	758.57	+0.03	195.39 ± 23.93	197.90 ± 27.36	0.565
20.15	PC(36:3)	C_44_H_83_NO_8_P^+^	784.63	784.58	+0.045	135.50 ± 23.95	133.31 ± 23.26	0.300
20.30	PC(34:1)	C_42_H_83_NO_8_P^+^	760.63	760.58	+0.05	149.14 ± 26.43	154.41 ± 26.00	0.235
20.32	PC(38:4)	C_46_H_85_NO_8_P^+^	810.68	810.60	+0.08	49.85 ± 13.02	49.02 ± 13.32	0.638
20.37	PC(36:2)	C_44_H_85_NO_8_P^+^	786.64	786.60	+0.04	168.87 ± 19.57	170.71 ± 25.72	0.635
20.43	PC(38:3)	C_46_H_87_NO_8_P^+^	812.68	812.62	+0.06	60.73 ± 26.71	55.31 ± 22.62	0.149
20.78	SM(d18:1/24:1(15Z))	C_47_H_94_N_2_O_6_P^+^	813.73	813.68	+0.05	132.91 ± 52.04	131.24 ± 59.93	0.704
20.79	SM(d18:0/22:1(13Z))	C_45_H_92_N_2_O_6_P^+^	787.72	787.67	+0.05	50.88 ± 40.64	44.42 ± 39.20	0.217

^a^Data are presented as mean ± SD. The *P* value was calculated by Mann-Whitney *U* test. LPC: lysophosphatidylcholine; PC: phosphatidylcholine; DG: diacylglycerol; SM: sphingomyelin.

**Table 5:** Identification and quantification of metabolites in human plasma using UHPLC-MS and electrospray ionization in negative-ion mode.

Retention time (min)	Common name	Formula of [M−H]^−^	Measured mass (*m/z*)	Theoretical mass (*m/z*)	Mass error (Da)	Non-DP	DP	*P*
0.64	unknown		291.08			122.96 ± 70.25^a^	136.14 ± 107.74	0.869
17.10	LPE(22:6)	C_27_H_43_NO_7_P^−^	524.27	524.28	<0.01	65.99 ± 30.35	61.52 ± 25.40	0.419
17.17	LPE(20:4)	C_25_H_43_NO_7_P^−^	500.27	500.27	<0.01	45.65 ± 20.26	40.26 ± 17.24	0.065
17.23	LPE(18:2)	C_23_H_43_NO_7_P^−^	476.27	476.28	<0.01	70.53 ± 34.49	65.03 ± 35.97	0.289
17.69	LPE(16:0)	C_21_H_43_NO_7_P^−^	452.27	452.28	<0.01	48.60 ± 17.28	46.68 ± 18.35	0.258
17.88	LPE(18:1)	C_23_H_45_NO_7_P^−^	478.29	478.29	<0.01	27.78 ± 18.78	24.01 ± 12.01	0.409
18.32	Eicosapentaenoic acid (C20:5)	C_20_H_29_O_2_^−^	301.21	301.22	<0.01	47.59 ± 31.47	48.31 ± 27.00	0.520
18.37	Linolenic acid (C18:3)	C_18_H_29_O_2_^−^	277.21	277.22	<0.01	58.12 ± 50.50	64.84 ± 64.66	0.495
18.64	Docosahexaenoic acid (C22:6)	C_22_H_31_O_2_^−^	327.23	327.23	<0.01	268.96 ± 121.50	294.55 ± 116.64	0.093
18.68	Free fatty acid (C16:1)	C_16_H_29_O_2_^−^	253.21	253.22	<0.01	83.04 ± 78.33	74.83 ± 63.94	0.901
18.77	Arachidonic acid (C20:4)	C_20_H_31_O_2_^−^	303.23	303.23	<0.01	161.10 ± 72.52	149.19 ± 77.86	0.306
18.86	Linoleic acid (C18:2)	C_18_H_31_O_2_^−^	279.23	279.23	<0.01	276.78 ± 191.57	279.87 ± 161.58	0.635
18.93	Docosapentaenoic acid (C22:5)	C_22_H_33_O_2_^−^	329.25	329.25	<0.01	32.17 ± 23.16	31.04 ± 18.84	0.749
19.04	Free fatty acid (C20:3)	C_20_H_33_O_2_^−^	305.25	305.25	<0.01	29.70 ± 19.08	29.47 ± 22.32	0.612
19.18	DG(30:1)	C_33_H_61_O_5_^−^	537.41	537.45	−0.04	17.87 ± 9.91	19.65 ± 9.45	0.176
19.27	Palmitic acid (C16:0)	C_16_H_31_O_2_^−^	255.23	255.23	<0.01	218.29 ± 140.27	209.29 ± 147.29	0.626
19.32	DG(30:0)	C_33_H_63_O_5_^−^	539.42	539.46	−0.04	11.81 ± 7.63	15.08 ± 16.86	0.440
19.36	Oleic acid (C18:1)	C_18_H_33_O_2_^−^	281.25	281.25	<0.01	402.57 ± 311.69	378.81 ± 270.00	0.875
19.55	unknown		393.27			63.50 ± 20.78	65.85 ± 22.13	0.641
19.57	MG(22:5)	C_25_H_39_O_4_^−^	403.30	403.28	+0.02	69.14 ± 27.41	67.50 ± 31.95	0.405
19.62	Free fatty acid (C17:0)	C_17_H_33_O_2_^−^	269.24	269.25	−0.01	20.24 ± 13.09	21.04 ± 14.72	0.862
19.86	unknown		364.49			54.03 ± 18.02	49.21 ± 15.44	0.054
19.89	Stearic acid (C18:0)	C_18_H_35_O_2_^−^	283.261	283.264	−0.003	243.29 ± 118.20	220.01 ± 101.87	0.212

^a^Data are presented as mean ± SD. The *P* value was calculated by Mann-Whitney *U*-test. LPE: lysophosphatidylethanolamine; DG: diacylglycerol, MG: monoacylglycerol; PE: phosphatidylethanolamine.

## References

[1] Lee JA, Lee JS, Kang BK (2011). Report on the Korean standard pattern identifications for stroke-III. *Korean Journal of Oriental Internal Medicine*.

[2] Go HY, Kim YK, Kang BK (2006). Report on the Korean standard differentiation of the symptoms and signs for the stroke-2. *Korean Journal of Oriental Physiology & Pathology*.

[3] Kim HJ, Bae HS, Park SU, Moon SK, Park JM, Jung WS (2011). Clinical approach to the standardization of oriental medical diagnostic pattern identification in stroke patients. *Evidence-Based Complementary and Alternative Medicine*.

[4] Jung WW, Lee WC (1998). The literature on “Dam-eum” resulted on stroke. *Dongguk Journal of the Institute of Oriental Medicine*.

[5] Kim SY, Lee JS, Kang BK (2009). Study on the relationship among Bi-Su type, obesity index and pattern identification in Korean stroke patients. *Korean Journal of Oriental Internal Medicine*.

[6] Kang JS, Kim DH, Shin HS (2009). The study on relationship of dampness-phlegm tongue diagnosis to hyperlippidemia in stroke patients. *The Journal of the Korea Institute of Oriental Medical Diagnosis*.

[7] Cha MH, Kim SY, Lim JH (2009). Study on the obesity and blood parameters differences between fire/heat and Qi-deficiency pattern identification/syndrome differentiation among acute stroke patient. *Korean Journal of Oriental Institute Medicine*.

[8] Lim JH, Ko MM, Lee JS (2010). Genetic association of SNPs located at PON1 gene with dampness and phlegm pattern identification among Korea stroke patients. *Korean Journal of Oriental Internal Medicine*.

[9] Ko MM, Kang BK, Lim JH, Lee MS, Cha MH (2012). Genetic association of NPY gene polymorphisms with dampness-phlegm pattern in Korean stroke patients. *Evidence-Based Complementary and Alternative Medicine*.

[10] Ko MM, Park TY, Lim JH, Cha MH, Lee MS (2012). WNT10B polymorphism in Korean stroke patients with Yin deficiency pattern. *Evidence-Based Complementary and Alternative Medicine*.

[11] Wu Y, Cun Y, Dong J (2010). Polymorphisms in PPARD, PPARG and APM1 associated with four types of traditional Chinese medicine constitutions. *Journal of Genetics and Genomics*.

[12] Lim JH, Ko MM, Lee JS (2011). Different level of plasma free hemoglobin between Qi-deficiency and fire heat among Korean stoke subjects. *Korean Journal of Oriental Physiology & Pathology*.

[13] Lim JH, Ko MM, Lee H (2012). Differential association of uncoupling protein 2 polymorphisms with pattern identification among Korean stroke patients: a diagnostic system in traditional Korean medicine. *Evidence-Based Complementary and Alternative Medicine*.

[14] Nicholson JK, Lindon JC (2008). Systems biology: metabonomics. *Nature*.

[15] Spratlin JL, Serkova NJ, Eckhardt SG (2009). Clinical applications of metabolomics in oncology: a review. *Clinical Cancer Research*.

[16] Claudino WM, Quattrone A, Biganzoli L, Pestrin M, Bertini I, Di Leo A (2007). Metabolomics: available results, current research projects in breast cancer, and future applications. *Journal of Clinical Oncology*.

[17] Qiu Y, Cai G, Su M (2009). Serum metabolite profiling of human colorectal cancer using GC-TOFMS and UPLC-QTOFMS. *Journal of Proteome Research*.

[18] Shah SH, Sun JL, Stevens RD (2012). Baseline metabolomic profiles predict cardiovascular events in patients at risk for coronary artery disease. *American Heart Journal*.

[19] Ciborowski M, Teul J, Martin-Ventura JL, Egido J, Barbas C (2012). Metabolomics with LC-QTOF-MS permits the prediction of disease stage in aortic abdominal aneurysm based on plasma metabolic fingerprint. *PLoS One*.

[20] Jung JY, Lee HS, Kang DG (2011). 1 H-NMR-based metabolomics study of cerebral infarction. *Stroke*.

[21] Gu Y, Lu C, Zha Q (2012). Plasma metabonomics study of rheumatoid arthritis and its Chinese medicine subtypes by using liquid chromatography and gas chromatography coupled with mass spectrometry. *Molecular Biosystems*.

[22] Wei H, Pasman W, Rubingh C (2012). Urine metabolomics combined with the personalized diagnosis guided by Chinese medicine reveals subtypes of pre-diabetes. *Molecular Biosystems*.

[23] Park TY, Lee JA, Cha MH (2012). The fundamental study for the standardization and objectification of pattern identification in traditional Korean medicine for stroke (SOPI-Stroke): an overview of phase I. *European Journal of Integrative Medicine*.

[24] Dunn WB, Broadhurst D, Begley P (2011). Procedures for large-scale metabolic profiling of serum and plasma using gas chromatography and liquid chromatography coupled to mass spectrometry. *Nature Protocols*.

[25] Kirstein-Grossman I, Beckmann JS, Lancet D, Miller A (2002). Pharmacogenetic development of personalized medicine: multiple sclerosis treatment as a model. *Drug News and Perspectives*.

[26] Trainer AH, Meiser B, Watts K, Mitchell G, Tucker K, Friedlander M (2010). Moving toward personalized medicine: treatment-focused genetic testing of women newly diagnosed with ovarian cancer. *International Journal of Gynecological Cancer*.

[27] Morrison KE (2011). Whole-genome sequencing informs treatment: personalized medicine takes another step forward. *Clinical Chemistry*.

[28] WHO (2007). *WHO International Standard Terminologies on Traditional Medicine in the Western Pacific Region*.

[29] Kang BK, Kang KK, Park SW (2007). The discrimination model for the pattern identification diagnosis of the stroke. *Korean Journal of Oriental Internal Medicine*.

[30] Ko MM, Park TY, Lee JA, Choi TY, Kang BK, Lee MS (2013). Interobserver reliability of pulse diagnosis using traditional Korean medicine for stroke patients. *The Journal of Anternative and Complementary Medicine*.

[31] Kang BK, Park TY, Lee JA (2012). Reliability and validity of the Korean standard pattern identification for stroke (K-SPI-Stroke) questionnaire. *BMC Complementary and Alternative Medicine*.

[32] Matsumoto T, Kobayashi T, Kamata K (2007). Role of lysophosphatidylcholine (LPC) in atherosclerosis. *Current Medicinal Chemistry*.

[33] Kuivenhoven JA, Pritchard H, Hill J, Frohlich J, Assmann G, Kastelein J (1997). The molecular pathology of lecithin:cholesterol acyltransferase (LCAT) deficiency syndromes. *Journal of Lipid Research*.

[34] Pietiläinen KH, Sysi-Aho M, Rissanen A (2007). Acquired obesity is associated with changes in the serum lipidomic profile independent of genetic effects—a monozygotic twin study. *PLoS One*.

[35] Barber MN, Risis S, Yang C (2012). Plasma lysophosphatidylcholine levels are reduced in obesity and type 2 diabetes. *PLoS One*.

[36] Kim HJ, Kim JH, Noh S (2011). Metabolomic analysis of livers and serum from high-fat diet induced obese mice. *Journal of Proteome Research*.

[37] Kim JY, Park JY, Kim OY (2010). Metabolic profiling of plasma in overweight/obese and lean men using ultra performance liquid chromatography and Q-TOF Mass spectrometry (UPLC-Q-TOF MS). *Journal of Proteome Research*.

